# Prevention of frailty in relation with social out-of-home activities in older adults: results from the Survey of Health, Ageing, and Retirement in Europe

**DOI:** 10.1007/s10433-024-00829-7

**Published:** 2024-11-16

**Authors:** Sandra A. Mümken, Enrique Alonso-Perez, Christine Haeger, Julie L. O’Sullivan, Qian-Li Xue, Sonia Lech, Wolfram J. Herrmann, Paul Gellert

**Affiliations:** 1 Institute of Medical Sociology and Rehabilitation Science, Charité – Universitätsmedizin Berlin, corporate member of Freie Universität Berlin and Humboldt Universität zu Berlin, Charitéplatz 1, 10117 Berlin, Germany; 2 Johns Hopkins Center On Aging and Health, Bloomberg School of Public Health, Johns Hopkins University, 2024 E. Monument Street, 700, Baltimore, MD USA; 3 Department of Psychiatry and Neuroscience, Charité – Universitätsmedizin Berlin, corporate member of Freie Universität Berlin and Humboldt Universität zu Berlin, Charitéplatz 1, 10117 Berlin, Germany; 4 Institute of General Practice and Family Medicine, Charité – Universitätsmedizin Berlin, corporate member of Freie Universität Berlin and Humboldt Universität zu Berlin, Charitéplatz 1, 10117 Berlin, Germany

**Keywords:** Frailty, Prevention, Out-of-home mobility, Social activities, Mediation analysis, Mixed models

## Abstract

Out-of-home mobility and social participation have been identified as resources to postpone frailty. We aim to examine the mediating role and specific contribution of social out-of-home activities in frailty prevention. Data from the Survey of Health, Ageing, and Retirement in Europe (SHARE) waves six (w6), seven (w7), and eight (w8) were used. Frailty was measured with the SHARE version of the Edmonton Frail Scale (EFS) with frailty states fit, pre-fail and frail. First, a mediation model with 13,456 fit participants aged ≥ 50 years in w6 was specified with social network size, loneliness (UCLA 3-Item Loneliness Scale), and lack of motivation (EURO-D) as predictors and number of performed social out-of-home activities in w7 as mediator variable on EFS-scores in w8. Age, education, gender, cohabitation, widowhood, urban environment, and country served as covariates. Second, we investigated the association of increasing social out-of-home activities from w6 to w7 with change in EFS-score from w6 to w8 using a linear mixed model with 17,439 participants in all frailty states. Direct effects of loneliness (w6) and lack of motivation (w6) on EFS-scores (w8) were partially mediated by social out-of-home activities (loneliness *ß* = 0.005; 95% CI = 0.003–0.008) and (lack of motivation *ß* = 0.014; 95% CI = 0.009–0.019). The linear mixed model revealed a significant effect of increasing social out-of-home activities (w6–w7) on reduction of EFS-scores (w8) (*ß* = − 0.21; 95% CI = − 0.29–0.04; *p* < 0.001). Social out-of-home activities appear to play a crucial role in frailty prevention, which could be used for future interventions.

**Supplementary Information:**

The online version contains supplementary material available at 10.1007/s10433-024-00829-7.

## Introduction

The age-related syndrome of frailty threatens older adults' independence, longevity, and quality of life (Clegg et al. 2013). Being frail raises the risk of adverse health outcomes such as institutionalization, depression, and mortality (Rockwood et al. 2004; Vermeiren et al. 2016), highlighting the importance of frailty prevention. Although no universal definition exists, frailty is commonly described as increased vulnerability towards external stressors that are age-related but distinct from normal ageing (Xue 2011). The two dominant concepts define frailty as either a physical phenotype including five criteria (i.e., weight loss, slowness, decreased strength, exhaustion, and activity reduction) (Fried et al. 2001) or within the concept of deficit accumulation by counting the number of age-related deficits and diseases (Mitnitski et al. 2005). Further approaches describe frailty as a multidimensional construct including the loss of physical, psychological, and social reserves (Gobbens et al. 2010). Thereby, the consent for applying multidimensional assessment tools in clinical practice and research is growing (Ambagtsheer et al. 2020; Sezgin et al. 2019).

The prevalence of frailty in older adults varies across frailty concepts and countries. For example, while one review found a pooled prevalence of 9.9% using the physical phenotype (Collard et al. 2012), another review using multidimensional definitions reported a pooled prevalence of 18.8% (Zhang et al. 2023). Across concepts, frailty is seen as a dynamic process that allows transition between different states, ranging from frailty-free to the development of first clinical manifestations of frailty. There exists a chance to delay the onset of frailty or revise its symptoms, especially when interventions occur early in frailty development (Travers et al. 2019).

Social and motivational factors play a significant role in the development and progression of frailty (Davies et al. 2021). Social factors identified in relation to frailty include participation in community activities, lack of social support, subjective feelings of loneliness (Bessa et al. 2021), and social network indicators like social network size (Hoogendijk et al. 2016), an objective marker of social isolation (Schutter et al. 2022). A recent study highlighted the importance of social activities for frailty status, demonstrating that participation in a higher number of diverse social activities was significantly associated with the reversion of frailty status (Jang et al. 2021). Moreover, longitudinal findings using SHARE data indicate that higher feelings of loneliness and social isolation were associated with onset of frailty (Jarach et al. 2021). Further, depressed mood elevates the danger of becoming frail (Prina et al. 2019) and often occurs in combination with motivation deficits (Grahek et al. 2019). Research suggests that the motivational component of depressive symptoms has the strongest connection with frailty, and therefore, a reversion of frailty might be achieved by addressing this specific component (Collard et al. 2017).

Different theories and possible pathways aim to explain the relationship between loneliness, social isolation, and frailty (Mehrabi & Béland 2020). These include the direct effect of loneliness and social isolation on physical health by influencing perceived stress, hypertension, and raising the risk for cardiovascular diseases and, therefore, frailty (Christiansen et al. 2021). Other theories refer to the stress-buffering effect of social contacts through providing support or fostering positive health behaviour (House et al. 1988). Additionally, reduced social engagement and social isolation are risk factors for cognitive decline, a symptom within multidimensional frailty definitions (Zunzunegui et al. 2003). In contrast, daily participation in communicative and intellectually demanding social activities such as playing cards or community-based physical activities was found to decrease the risk of frailty (Sun et al. 2022). Explanations for the protective influence of community activities against failty include their socially integrative and identity-creating effect (Ang 2018), and their impact on self-rated health (Leone and Hessel 2016). According to the model of life-space constriction (Xue et al. 2007), most social activities require regular out-of-home mobility within a person’s life-space, defined as the in- and outdoor areas a person moves through in daily life (Sawyer et al. 2010). Reduced social activities that are performed outside one’s home (social out-of-home activities) may be associated with constricted life-space mobility, which, in turn, is associated with loneliness (Petersen et al. 2015), frailty (Portegijs et al. 2016), and mortality (Kennedy et al. 2017). Therefore, the question arises of whether participation in social out-of-home activities could be a promising approach for preventing or even reversing frailty by facilitating out-of-home mobility and social interactions, a mechanism that is currently not well understood.

First, our study aims to identify the mediating role of social out-of-home activities within the relationship between frailty risk factors—such as one's social network, loneliness, and lack of motivation—and frailty status over time within a sample of community-dwelling older adults. We use a multidimensional definition of frailty that includes problems and loss of reserves in multiple domains, making the individual vulnerable to environmental challenges (Strawbridge et al. 1998). Second, we investigate how an increase in social out-of-home activities impacts frailty after 5 years. For both analyses, we hypothesize a protective effect of social out-of-home activities.

## Methods

### Data source and study participants

This study analysed three consecutive waves from the Survey of Health, Ageing, and Retirement in Europe (SHARE) between 2015 and 2020. SHARE was established in 2004 and is the largest panel survey collecting health, financial, and social data from respondents aged 50 and over across Europe (Börsch-Supan et al. 2013). Ethical approval for SHARE was received from the University of Mannheim and is continuously updated by the Max Planck Society (Braun 2021). The survey interviews were conducted as computer-assisted personal interviews (CAPI) by trained interviewers and were switched to telephone-based interviews (CATI) in 2020 due to the COVID-19 pandemic.

**Fig. 1:**
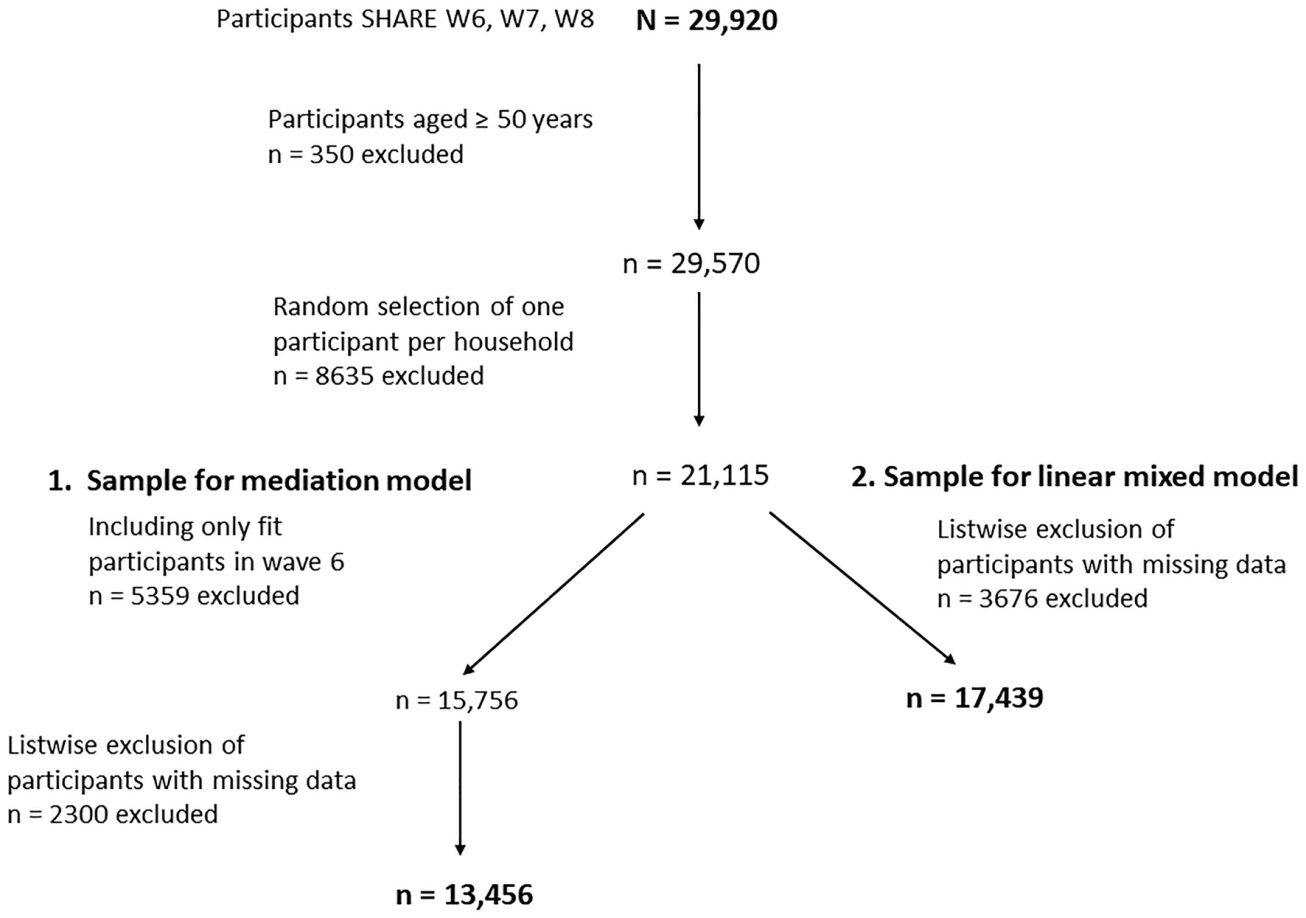
Flowchart of included participants: SHARE = Survey of Health, Ageing, and Retirement in Europe. *N* = total sample size of participants who participated sequent in SHARE waves 6, 7, and 8; sample size for the mediation model *n* = 13,456; sample size for the mixed model *n* = 17,439; countries included in both analyses were Austria, Germany, Sweden, Spain, Italy, France, Denmark, Greece, Switzerland, Belgium, Israel, Czech Republic, Poland, Luxemburg, Slovenia, Estonia, and Croatia

We selected two study samples drawn from SHARE participants of wave 6 (2015) who completed the follow-ups in wave 7 (2017) and wave 8 (2020), generating a total sample of *N* = 29,920 (Fig. 1). By selecting this sequence of waves, we ensured that the mediator social out-of-home activities was not impacted by the lockdown restrictions during the COVID-19 pandemic. We selected those aged ≥ 50 years (*n* = 29,570). To achieve independence of observations, one person per household was randomly selected, leading to a sample of *n* = 21,115 (exclusion of *n* = 8635). To derive the study sample for our first research question (mediation model), only participants classified as fit (frailty-free) in wave 6 were selected (*n* = 15,756), leading to the exclusion of *n* = 5359 participants. After excluding *n* = 2300 participants with missing data for frailty status (wave 8), predictor variables and covariates (wave 6), and mediator variable (wave 7), the final sample included *n* = 13,456 participants. For the second research question (linear mixed model), we included all participants with valid data regardless of their frailty status at wave 6 (*n* = 17,439). We excluded *n* = 3676 participants due to missing data for the mixed model. Both selected samples included participants from 17 countries: Austria, Germany, Sweden, Spain, Italy, France, Denmark, Greece, Switzerland, Belgium, Israel, Czech Republic, Poland, Luxemburg, Slovenia, Estonia, and Croatia.

## Measures

### Outcome: frailty

The Edmonton Frail Scale (EFS) measures frailty as a multidimensional construct using nine different health domains: (1) cognition, (2) mood, (3) functional independence, (4) nutrition, (5) social support, (6) continence and (7) functional performance, (8) medication use, and (9) general health status. The EFS-score ranges from 0 to 17 points, where 17 represents the highest level of frailty (Rolfson et al. 2006), and is recommended as an accurate assessment tool for multidimensional frailty among community-dwelling older adults (Ambagtsheer et al. 2020). We used a modified version that had been validated and compared to seven other frailty scales using SHARE data. The modified version consists of 11 items to evaluate frailty symptoms (range 0–17), showing no ceiling effects and good feasibility (Theou et al. 2013). In the present study, cases with ≤ 2 missing items on the EFS were considered valid and missing values scored 0 (Theou et al. 2013). To classify the different frailty states of fit (i.e., frailty-free), vulnerable (i.e., first signs of frailty), and frail (i.e., manifestation of frailty), the following cut of points were used: 0–3 points = fit (He et al. 2020), 4–7 points = vulnerable, and 8–17 points = frail (Theou et al. 2013). For the second research question, a metric variable (0–17) was calculated to represent the change in EFS between wave 6 and wave 8, where negative values indicated a decrease in the frailty symptoms, whereas positive values indicated an increment.

### Predictor 1: social network size

Social network size (SN size) as single-objective indicator of social network structure and social isolation was chosen as a predictor. SN size was measured by asking each participant to name their close social contacts, defined as “someone with whom you discuss important personal matters”. Participants were asked to name a maximum of seven persons, but this boundary is only mentioned when a participant reaches this limit. Therefore, the SN size score ranges from 0 to 7 persons (Litwin et al. 2015).

### Predictor 2: loneliness

Loneliness is a subjective indicator of a person's experienced quality in social relationships (Schutter et al. 2022). Loneliness was measured using the validated 3-Item Loneliness Scale, a short version of the Revised UCLA Loneliness Scale (Hughes et al. 2004). The UCLA 3-Item Loneliness Scale asks about experienced feelings of loneliness, social isolation, and lack of companionship with the response options of hardly never (1 point), sometimes (2 points), and often (3 points). Points for each question are added up, creating a score with a range of 3–9 points, where higher values indicate greater feelings of loneliness, and people can be grouped not lonely (3–5) and lonely (6–9) (Steptoe et al. 2013).

### Predictor 3: lack of motivation

Lack of motivation was measured with four items (pessimism, interest, concentration, and enjoyment) of the EURO-D depression scale representing the factor motivation (Maskileyson et al. 2021). All items have dichotomized response formats (any = 1 and none = 0). To derive a scale (0–4) where high values indicate a higher lack of motivation, the following questions were included: 1. “What are your hopes for the future?” (Pessimism, EURO 2); 2. “In the last month, what was your interest in things?” (Interest, EURO 6); 3. “How is your concentration? For example, can you concentrate on a television program, film, or radio program?” (Concentration, EURO 10) and 3. “What have you enjoyed doing recently?” (Enjoyment, EURO 11). The sum of all four dichotomized items was calculated, and participants with more than two missing items were excluded.

### Mediator: social out-of-home activities

The number of social out-of-home activities was calculated as the sum score of four social activities that usually require leaving the house and are assessed within the questionnaire module “Activities (AC)” of SHARE. The following questions about social activities in the last year that could be answered yes or no were used: In the last year, have you done one of the following activities? 1. “Done voluntary or charity work?” 2. “Attended an educational or training course?” 3. “Gone to a sports or other kind of social club?” and 4. “Taken part in a political or community-related organization?” The sum of all conducted social activities was calculated, receiving a score ranging from 0 to 4, and only participants who answered to all four activities were included as valid cases. For the second research question, a binary variable indicating an increase in performed social out-of-home activities between wave 6 and wave 7 was created.

### Covariates

Sociodemographic factors that are known to be associated with frailty, i.e., age, sex, urban living environment, education, country lived in, and cohabitating (Collard et al. 2012; Manfredi et al. 2019) were drawn from wave 6. Education was categorized as low (ISCED 0–2), medium (ISCED 3–4), and high (ISCED 5–8) according to the International Standard Classification of Education (ISCED) from 2011 (Schneider 2013). Cohabitating was defined as living with someone or alone, and widowing was defined as having experienced the loss of a married partner. The urban living environment was characterized as living in a large city or not and was subjectively assessed by the respondents. Further descriptive variables included the number of limitations in activities of daily living (iADL) that are expected to last longer than 3 months and number of chronic diseases.

## Analysis

### Mediation analysis

First, descriptive statistics were calculated for predictor, mediator, and covariates. To investigate the mediating role of social out-of-home activities as a potential pathway for frailty prevention, a longitudinal simple mediation model was specified with SN size, loneliness, and lack of motivation measured in wave 6 as predictors, social out-of-home activities measured in wave 7 as the mediator, and frailty measured in wave 8 as the outcome. We created dummy variables for each country included in the sample. Age, sex, education, and urban living environment and country dummy variables served as covariates measured in wave 6. To control for frailty status in wave 6, only participants classified as fit robust (EFS-score 0–3) were included in the analysis. All analyses were conducted with SPSS 27. To receive the post hoc estimates of the indirect effect, our simple mediation model was entered three times with swapping order of the independent variable and the covariates. By applying this strategy, the models are identical, but the estimate for the indirect effects will be estimated for each of the three predictors individually. The mediation models were tested with ordinary least square regression and bootstrap sampling with replacement to derive fit 95% confidence intervals (CI) using the PROCESS Macro of Andrew F. Hayes (Hayes 2022). For the bootstrapping procedure, we used 10,000 resamples and a fixed random seed (5235). Applying this method, indirect mediation effects are significant if the value 0 is not included in the estimated 95% bootstrapped CIs (Igartua & Hayes 2021). To estimate how much of the total effect of all predictor variables was mediated by the number of social out-of-home activities, we calculated the proportion mediated as the quotient of the indirect effect and total effect (indirect/total effect). Figures were created using PowerPoint 2016.

### Mixed model for predicting frailty

To examine the relation of social out-of-home activities and frailty across waves, we specified a linear mixed model with individuals (level 1) nested within countries (level 2). Change scores were used to measure social out-of-home activities and frailty change. For the linear mixed model, we used the restricted maximum likelihood (REML) estimator to test whether an increase in social out-of-home activities between wave 6 and wave 7 was associated with changes in the EFS. Therefore, the difference in EFS-scores between wave 8 and wave 6 was selected as outcome variable. Subsequently, education was entered as categorical variable together with age, gender, cohabitating, social network size, feelings of loneliness, lack of motivation, and the sum of social out-of-home activities in wave 6 as fixed effects. Participants’ countries (dummy variables) were set as a random effect, while EFS-score measured at wave 6 was controlled for. Due to missing information on social out-of-home activities in wave 6, a total of 3676 cases were excluded, leading to a final sample of *n* = 17,439 for the mixed model (Fig. 1).

## Results

Of the 21,115 randomly selected participants per household in wave 6 valid information for computing EFS-scores could be obtained for 21,011. Of these 21,011 participants, 74.99% were fit, 21.64% vulerable, and 3.37% frail. Supplement 1 provides further information about the participant’s frailty status in wave 6 and wave 8.

Sample characteristics at the first time point (wave 6) of the analysis sample for mediation analysis (*n* = 13,456; all in fit frailty state) and the analysis sample for the linear mixed model (*n* = 17,439; all frailty states) are reported in Table 1. Participants included in the mediation analysis were between 50 and 94 years old with a mean age of 65.49 years, 58.05% of them were women. About 66.40% lived in an urban environment, 73.38% were cohabitating with someone, 15.04% were widowed, had 1.36 chronic diseases, and 0.60 iADL limitations on average. Participants experienced on average a low level of loneliness (M = 3.68), a low lack of motivation (M = 0.28), and had an average SN size (M = 2.82). Education was classified as high for 27.45%, medium for 43.82%, and low for 28.73%. The mean age of the *n* = 17,439 participants included in the mixed model was 67.37 years, 60.96% were women, 66.37% lived in an urban environment, and 70.91% were cohabitating, 18.05% were widowed. Participants had 1.74 chronic diseases and 0.28 iADL limitations on average. On average participants experienced a low level of loneliness (M = 3.89), a low lack of motivation (M = 0.28), and had an average SN size (M = 2.79). Education was classified as high for 32.57%, medium for 42.26%, and low for 25.17%. Concerning social out-of-home activities, 16.26% increased their engagement.

**Table 1:** Descriptive characteristics

Variable	*n* = 13,456 (mediation model)		*n* = 17,439 (linear mixed model)	
	*n*	*%*	*n*	*%*
Gender (female)	7811	58.05	10,630	60.96
Cohabitating with someone	9874	73.38	12,376	70.91
Widowed	2025	15.04	3148	18.05
Urban	8935	66.40	11,574	66.37
Education				
High	3866	27.45	5680	32.57
Medium	5897	43.82	7370	42.26
Low	3693	28.73	4389	25.17
Increased social out-of-home activities from SHARE wave 6 to SHARE wave 7 (yes)			2836	16.26

	*M*	*SD*	*M*	*SD*
Age (years)	65.94	8.66	67.37	8.81
Number of chronic diseases	1.36	1.25	1.74	1.52
Number of iADL limitations (0–9)	0.60	0.30	0.28	0.93
Social network size (0–7)	2.82	1.56	2.79	1.55
Loneliness (3–9)	3.68	1.10	3.89	1.32
Lack of motivation (0–4)	0.28	0.58	0.40	0.73
Sum of social out-of-home activities SHARE wave 7	0.82	0.96	0.86	0.90

*N* = 29,920; *n* = 13,456 fit participants in 2015 (SHARE wave 6) included in mediation analysis; *n* = 17,479 participants in 2015 (SHARE wave 6) included in the mixed model; SHARE = Survey of Health, Ageing, and Retirement in Europe; sum of four social out-of-home activities performed last year: 1. done voluntary or charity work, 2. attended an educational or training course, 3. gone to a sport, social, or other kind of club, and 4. taken part in a political- or community-related organization; iADL = instrumental activities of daily living

**Fig. 2:**
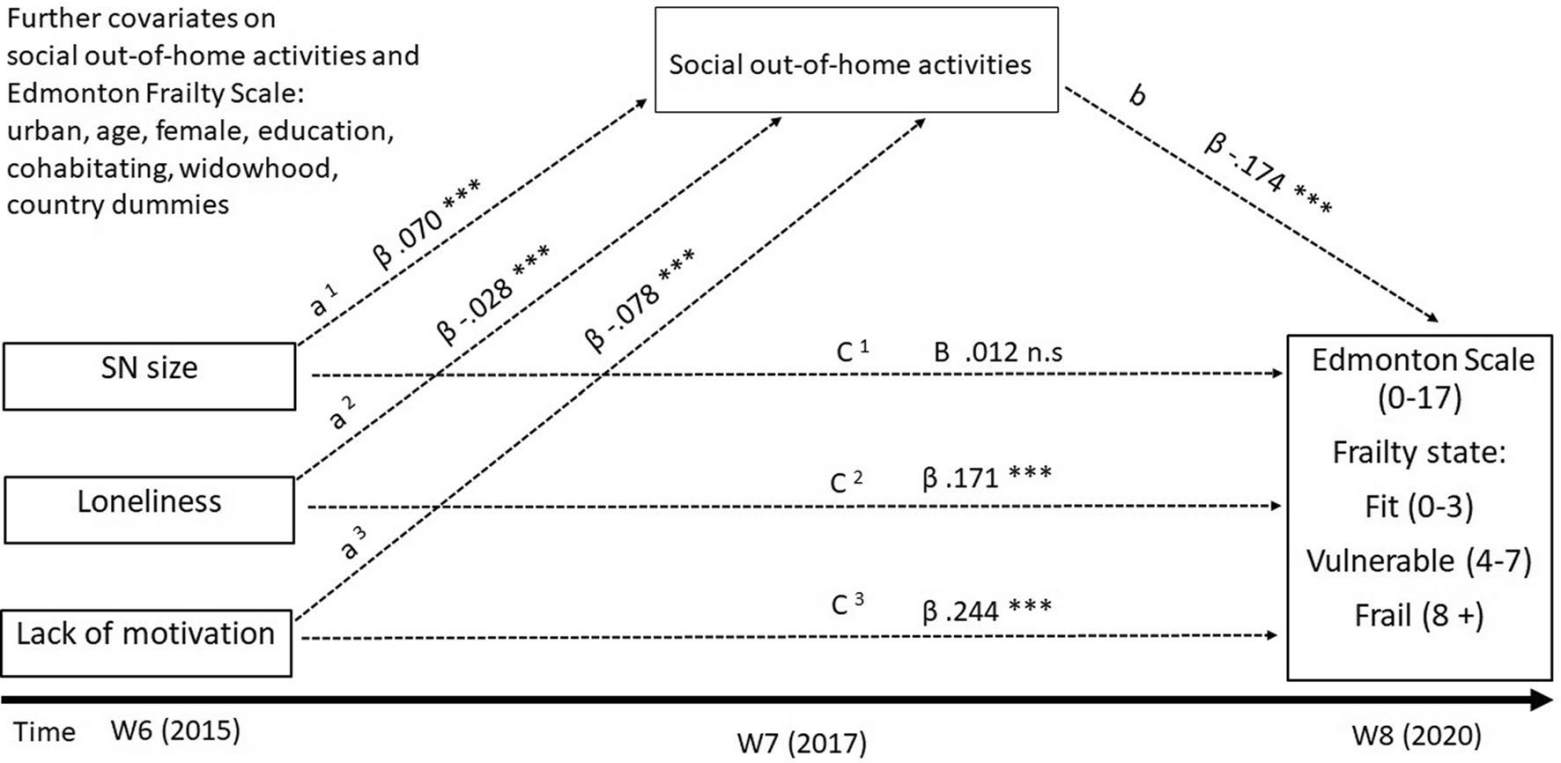
Mediation model: 95% CI = Confidence interval 95% with lower limit/upper limit; ***significant *p* < 0.001; n.s. = not significant. Three consecutive waves of the Survey of Health, Ageing, and Retirement in Europe (SHARE) were used: wave 6 (W6), wave 7 (W7), and wave 8 (W8); indirect effect of social network size (SN size) on Edmonton Frail Scale (EFS) mediated via social out-of-home activities (a1 *b): *ß* = − 0.012; 95% CI = − 0.015 to − 0.009; indirect effect of loneliness on EFS via social out-of-home activities (a2*b): *ß* = 0.005; 95% CI = 0.003–0.008; indirect effect of lack of motivation on EFS mediated via social out-of-home activities (a3 *b): *ß* = 0.014; CI 95% = 0.009–0.019

Results of the mediation analysis between the predictors SN size, loneliness, and lack of motivation through social out-of-home activities are presented in Table 2. Coefficients of investigated mediation paths are displayed in Fig. 2.

**Table 2:** Statistical results of the longitudinal mediation analysis

n = 13,456	Coefficient	95% CI	*p* value	Proportion mediated %
Predictor variables				
Social network size				
Total effect	− 0.000	− 0.019, 0.019	0.993	n.a
Direct effect	0.012	− 0.008, 0.032	0.238	
Indirect effect (95% bootstrap CI)	− 0.012	− 0.015, − 0.009	–	
Effect on social out-of-home activities (a)	0.070	0.059, 0.079	< 0.001	
Effect social out-of-home activities on frailty (b)	− 0.174	− 0.206, − 0.141	< 0.001	
Loneliness				
Total effect	0.176	0.146, 0.207	< 0.001	2.841
Direct effect	0.171	0.140, 0.202	< 0.001	
Indirect effect (95% bootstrap CI)	0.005	0.003, 0.008	–	
Effect on social out-of-home activities (a)	− 0.028	− 0.042, − 0.061	< 0.001	
Effect social out-of-home activities on frailty (b)	− 0.174	− 0.206, − 0.141	< 0.001	
Lack of motivation				
Total effect	0.258	0.198, 0.317	< 0.001	5.426
Direct effect	0.244	0.185, 0.304	< 0.001	
Indirect effect (95% bootstrap CI)	0.014	0.009, 0.019	–	
Effect on social out-of-home activities (a)	− 0.078	− 0.101, − 0.055	< 0.001	
Effect social out-of-home activities on frailty (b)	− 0.174	− 0.206, − 141	< 0.001	

Mediator variable: Social out-of-home activities (sum 0–4); only participants that were classified fit in SHARE wave 6 (2015) were included; analysis adjusted for age, sex, education, cohabitation, living environment, widowhood, and country dummy variables; Austria = reference country; 95% CI = 95% confidence interval; to test significance of indirect effects 95% bootstrap confidence interval with 10,000 repetitions was used; (a) = effect predictor on mediator and (b) = effect mediator on outcome; n.a. = not available due to insignificant direct effect

No significant total effect (*ß* = − 0.000; 95% CI = − 0.019–0.019; *p* = 0.993) and no significant direct effect (*ß* = 0.012; 95% CI = − 0.008–0.032; *p* = 0.238) of SN size wave 6 on frailty wave 8 were found. In contrast, we discovered significant effects between SN size wave 6 and the mediator social out-of-home activities wave 7 (*ß* = 0.070; 95% CI = 0.059–0.079; *p* < 0.001). For the mediator variable social out-of-home activities in wave 7, a significant negative effect on frailty status in wave 8 (*ß* =− 0.174; 95% CI = − 0.206 to − 0.141; *p* < 0.001) was found. The indirect effect of SN size wave 6 meditated through the number of social out-of-home activities in wave 7 on frailty in wave 8 was found to be significant (*ß* = − 0.012; 95% CI = − 0.015 to − 0.009).

The predictor loneliness wave 6 and the included mediator social out-of-home activities revealed a significant total effect on frailty wave 8 (*ß* = 0.176; 95% CI = 0.146–0.207; *p* < 0.001). This total effect constituted of a significant direct effect of loneliness wave 6 on frailty in wave 8 (*ß* = 0.171; 95% CI = 0.140–0.202; *p* < 0.001) and a significant indirect effect through the number of social out-of-home activities in wave 7 (*ß* = 0.005; 95% CI = 0.003–0.008). A proportion of 2.841% of the total effect of loneliness in wave 6 on frailty in wave 8 was mediated by out-of-home activities in wave 7, revealing a partial mediation (proportion mediated). Additionally, loneliness in wave 6 significantly negatively affected the mediator social out-of-home activities in wave 7 (*ß* = − 0.028; 95% CI = − 0.042 to − 0.061; *p* < 0.001). For the third predictor, lack of motivation in wave 6, a significant total effect (*ß* = 0.258; 95% CI = 0.198–0.317; *p* < 0.001) and a significant direct effect on frailty in wave 8 (*ß* = 0.244; 95% CI = 0.185–0.304; *p* < 0.001) were demonstrated. The effect of lack of motivation in wave 6 on the mediator social out-of-home activities in wave 7 was established as significant and negative (*ß* = − 0.078; 95% CI = − 0.101 to − 0.055; *p* < 0.001). Further, a significant indirect effect of lack of motivation wave 6 through social out-of-home activities wave 7 on frailty wave 8 (*ß* = 0.014; 95% CI = 0.009–0.019) explained 5.426% (proportion mediated) of the relationship between lack of motivation wave 6 and frailty wave 8.

Table 3 shows the results of the linear mixed model, characterizing the impact of variables at wave 6 on EFS-scores changes between waves 6 and 8 (*n* = 17,439). The partially adjusted model (model 1) shows how both social out-of-home activities in wave 6 (*ß* = − 0.16; 95% CI = − 0.19 to − 0.13; *p* < 0.001) and an increase in them between waves 6 and 7 (*ß* = − 0.22; 95% CI = − 0.30 to − 0.15; *p* < 0.001) significantly reduced the frailty symptoms between waves 6 and 8.

**Table 3:** Results of the linear mixed model on EFS-score changes between waves 6 and 8

Variable at baseline (wave 6)	Model 1 (partially adjusted)			Model 2 (fully adjusted)		
	Β	95% CI	*P* value	β	95% CI	*P* value
Sum of social out-of-home activities	− 0.16	− 0.19, − 0.13	< 0.001	− 0.15	− 0.18, − 0.11	< 0.001
Increase social out-of-home activities SHARE wave 6 to wave 7	− 0.22	− 0.30, − 0.15	< 0.001	− 0.21	− 0.29, − 0.14	< 0.001
Social network size				− 0.00	− 0.02, 0.02	0.719
Loneliness				0.08	0.06, 0.10	< 0.001
Lack of motivation				0.14	0.10, 0.18	< 0.001

Both models are adjusted for age, sex, education, cohabitation, living environment, widowhood, and frailty status at baseline; country dummy variables were entered as random effect; Austria = reference country *n* = 17,439; 95% CI = 95% Confidence interval

Model 2 further adjusts for social network, loneliness, and motivation. The sum of social out-of-home activities performed in wave 6 (*ß* = − 0.15; 95% CI = − 0.18 to − 0.11; *p* < 0.001) as well as the increase in social out-of-home activities from wave 6 to wave 7 (*ß* = − 0.21; 95% CI = − 0.29 to − 0.14; *p* < 0.001) were significantly associated with negative values on the frailty change score. Further, we found that loneliness (*ß* = 0.08; 95% CI = 0.06–0.10; *p* < 0.001) and lack of motivation (*ß* = 0.14; 95% CI = 0.10–0.18; *p* < 0.001) significantly increased the frailty symptoms from wave 6 to wave 8. In contrast, SN size in wave 6 had no significant association with EFS-scores changes in wave 8 (*ß* = − 0.00; 95% CI = − 0.02 to 0.02; *p* = 0.719). Complete results of the fully adjusted mixed model are displayed in supplement 2. Sensitivity analyses revealed significant cross-sectional associations of loneliness, lack of motivation, low education, and sum of social out-of-home activities with frailty satus in wave 6, wave 7, and wave 8. In wave 8, widowhood was significantly associated with frailty status too. The increase in social out-of-home activities from wave 6 to 7 was not significantly associated with frailty satus in wave 7 or wave 8. A longitudinal sensitivity analysis with the change score of frailty from wave 6 to wave 7 as outcome demonstrated a significant association with the increase in social out-of-home activities from wave 6 to wave 7. Complete results of the sensitivity analyses are presented in supplement 3.

## Discussion

We contribute to the existing research gap on frailty prevention by investigating the relationship between SN size, loneliness, and lack of motivation in relation to social out-of-home activities on the development of frailty using an adapted version of the EFS within three consecutive waves of the SHARE survey. By applying a longitudinal mediation model, we found that the direct effects of loneliness and lack of motivation on EFS-scores were partially mediated by performed social out-of-home activities. Further, we found a significant association between increasing participation in social out-of-home activities and a reduction of frailty symptoms using a linear mixed model. This confirms our hypothesis of identifying a protective effect of social out-of-home activities in frailty development.

First, the results of our longitudinal mediation analysis demonstrate a significant direct effect of loneliness on the development of frailty in 5 years and confirm the findings of other longitudinal studies using multidimensional and physical phenotype frailty definitions (Bessa et al. 2021; Jarach et al. 2021). In contrast, we did not find a direct effect of SN size as a single-objective indicator of social isolation, while other longitudinal studies did, using an index to assess social isolation (Davies et al. 2021). This was contrary to our expectation, as associations of SN size with frailty and adverse health outcomes have been found (Hoogendijk et al. 2016; Schutter et al. 2022). Our results allow the direct comparison of loneliness and SN size and contribute to a defined research gap (Mehrabi & Béland 2020). In order to not oversee a potential causal mediation effect (MacKinnon et al. 2007), we specified SN size as a predictor in our mediation analysis and found a small but significant indirect effect of social out-of-home activities in the relationship between SN size and frailty which should not be overestimated. Further our results demonstrate the negative impact of loneliness and lack of motivation on social participation and confirm other studies (McHugh Power et al. 2019). Second, a protective effect of social out-of-home activities is demonstrated by the significant indirect effect of social out-of-home activities within the relationship between loneliness and frailty and lack of motivation and frailty found in our mediation model. Although the proportion mediated through social out-of-home activities was relatively small in both cases, our results meet our expectations as indirect effects are nearly always small (Walters 2019).

The results of our mixed model confirm the protective effect of social out-of-home activities against the development of frailty in two ways: First, the number of social out-of-home activities participated in wave 7 as well as the increment of social out-of-home activities from wave 6 to wave 7 significantly reduced the worsening of frailty between wave 6 and wave 8 in the adjusted and fully adjusted model. Both results indicate that social out-of-home activities contribute to long-term frailty prevention and are in line with other research that identified the protective effect of social participation on depression and chronic conditions (Ang 2018). Moreover, our mixed model revealed that loneliness and the lack of motivation are positively associated with the development of frailty over time. Our results were confirmed by cross-sectional sensitivity analyses on frailty for each wave. Concerning the increment of social out-of-home activities between wave 6 and wave 7, sensitivity analysis exposed a significant association with the change of frailty between wave 6 and wave 7 in the direction that an increase in social out-of-home activities was significantly associated with the reduction of frailty in wave 7. These results demonstrate that fostering social out-of-home activities is an important strategy for preventing and revising frailty (Travers et al. 2019).

Other studies that investigated the longitudinal effects of social factors and participation in social activities on frailty both applied a different study design using two measurement points (Jang et al. 2021; Sun et al. 2022). One study focused on the frequency of participating in different social activities performed inside and outside the house, like playing cards, using the internet or caring for a distant relative and demonstrated that a daily frequency of participation in those activities is associated with reversing frailty progression (Sun et al. 2022). In contrast, our study focused on the participation in four social activities that can be seen as surrogate parameters for participants’ out-of-home mobility in wave 7.

Thus, the context of life-space mobility can be considered as a possible explanation for the mediating role of social out-of-home activities identified in our study. Participation in fewer social out-of-home activities can be seen as an early sign of life-space constriction and reduced physiological reserves, which are associated with lower autonomy to move outdoors (Portegijs et al. 2016), frailty (Xue et al. 2007), and mortality (Kennedy et al. 2017). Further explanations include, that social out-of-home activities strengthen the maintenance of social contacts despite the tendency of shrinking social networks in older age and contribute to the exchange of functional and emotional social support and health information, which may influence the onset of frailty (House et al. 1988). Additionally, participation in social out-of-home activities supports a greater sense of community belonging and the social identity of older adults (Michalski et al. 2020), which may improve frailty through their association with better self-rated and objective health (Leone & Hessel 2016).

### Strengths and limitations

A particular strength of our study is that we used a longitudinal study design to conduct a mediation analysis for the three predictors (SN size, loneliness, and lack of motivation) and the mediator social out-of-home activities that, due to its temporal consecutive order of measurement time points, allowed the identification of causal associations. Moreover, we used two statistical methods to investigate the meaning of social out-of-home activities for frailty prevention among SHARE survey participants. We needed to exclude 28.86% of the total sample to achieve independence of observations (i.e., cohabitating participants). Missing values were low with 7.69% in the mediation model and 12.29% in the mixed model, respectively. Even though these numbers were small, this may limit the generalizability of our results. Nonetheless, we were still able to include an adequate sample size of more than 13,000 participants located in 17 different European countries in both models.

One limitation of our study is that we did not include giving support to family members or friends living outside ones house as an out-of-home activity in our analysis as other studies did (Sun et al. 2022). We chose this as we wanted to focus on voluntary activities that foster out-of-home mobility that can be targeted in interventions. Furthermore, we only included SN size and did not use a composite index summarizing diverse SN aspects which assesses social isolation more integrally. On the other hand, our results now specifically refer to SN size and are not blurred (Schutter et al. 2022). Additionally, our outcome (EFS) was partly assessed in 2020 and might be influenced by the COVID-19 pandemic. We consider this influence to be small, as the results of the first SHARE Corona survey indicated no difference in self-rated and mental health between participants in 2019 and those participating in 2020 (SHARE-ERIC 2021).

A key strength of our study is the use of the EFS, a validated frailty scale in SHARE that best predicts all-cause mortality and aligns more closely with the original EFS than the widely used SHARE frailty phenotype to its real-life pendant (Theou et al. 2013). Otherwise, our results are not easily comparable to other studies conducted with SHARE data using the fried frailty phenotype, which is more prone to ceiling effects (Theou et al. 2014). Thus, referring to our results, we can not have overestimated the effect of social out-of-home activities on the development of frailty.

### Implications for future research

Our research reveals the importance of social out-of-home activities for frailty prevention and questions arise that should be part of future investigations. First, the underlying mechanisms of how social out-of-home activities contribute to frailty prevention remains unclear. We discussed possible effects of the mobility component of social out-of-home activities on frailty but future research should clarify the relationship between personal and social factors, out-of-home mobility and frailty using specific measures for out-of-home mobility. Further, factors such as the perceived autonomy to move outdoors or sense of community belonging should be considered as mechanisms that contribute to the protective effect of social out-of-home activities against frailty development. Additionally, future studies should investigate the influence of other social network components such as social network type (diverse, family-focused, or friend-focused) or perceived social support on frailty and social out-of-home activities.

## Conclusion

Interventions to prevent or reverse frailty should target increasing or stabilizing participation in social out-of-home activities to tackle loneliness and lack of motivation as frailty risk factors. Further, increasing social out-of-home activities has a significant protective effect on frailty development.

## Supplementary Information

Below is the link to the electronic supplementary material.

Supplementary file 1 (DOCX 29 kb)

Supplementary file 2 (DOCX 27 kb)

Supplementary file 3 (DOCX 38 kb)

## Data Availability

This study uses data of the Survey of Health, Ageing, and Retirement in Europe (SHARE). SHARE data are publicly available (https://share-eric.eu/data/, February 2024)
