# Supplementary material for: Prevention of frailty in relation with social out-of-home activities in older adults: results from the Survey of Health, Ageing, and Retirement in Europe
Source: Eur J Ageing. 2024 Nov 16;21(1):35. doi: 10.1007/s10433-024-00829-7 (PMC11569102; doi:10.1007/s10433-024-00829-7)
Supplement: Supplementary file 1 — (DOCX 29 kb) [file 10433_2024_829_MOESM1_ESM.docx]

# Supplement 1

Frailty state of different samples drawn from SHARE wave 6 (2015)

| **Frailty state** | **n** | **%** |
| --- | --- | --- |
| n = 29,570 | | |
| Fit | 22,367 | 75.67 |
| Vulnerable | 6139 | 20.76 |
| Frail | 873 | 2.95 |
| Missing | 182 | 0.62 |
| n = 21,115 (including one person per household) | | |
| Fit | 15,756 | 74.62 |
| Vulnerable | 4547 | 21.53 |
| Frail | 708 | 3.35 |
| Missing | 104 | 0.49 |
| n = 17,439 (mixed model) | | |
| Fit | 13,433 | 77.03 |
| Vulnerable | 3557 | 20.39 |
| Frail | 449 | 2.57 |
| Missing | --- | --- |
| Note: SHARE = Survey of Health, Aging, and Retirement in Europe; participants over 50 years of SHARE wave 6with ≤ 2 missing items on Edmonton Frail Scale were included; To archive independency of observation only one participant per household was selected; please note percentages have been rounded | | |

Frailty state of participants in SHARE wave 8 (2020)

| **Frailty state** | **n** | **%** |
| --- | --- | --- |
| n = 13,456 | | |
| Fit | 10,999 | 81.74 |
| Vulnerable | 2204 | 16.38 |
| Frail | 253 | 1.88 |
| Missing | --- | --- |
| n = 17,439 | | |
| Fit | 12,185 | 69.87 |
| Vulnerable | 4183 | 23.99 |
| Frail | 1071 | 6.14 |
| Missing | --- | --- |
| Note: SHARE = Survey of Health, Aging, and Retirement in Europe; Participants with missing items ≤ 2 on Edmonton Frail Scale were included; 13,456 participants were included in the mediation analysis; 17,349 participants were included in the analysis of the mixed model; please note percentages have been rounded | | |
