# Supplementary material for: Prevention of frailty in relation with social out-of-home activities in older adults: results from the Survey of Health, Ageing, and Retirement in Europe
Source: Eur J Ageing. 2024 Nov 16;21(1):35. doi: 10.1007/s10433-024-00829-7 (PMC11569102; doi:10.1007/s10433-024-00829-7)
Supplement: Supplementary file 2 — (DOCX 27 kb) [file 10433_2024_829_MOESM2_ESM.docx]

Appendix 2 Complete results of the fully adjusted mixed model

| Predictor | β | SE | 95% CI | 95 % UCI | *P* value |
| --- | --- | --- | --- | --- | --- |
| SHARE Wave 6 (2015) |  |  |  |  |  |
| **Age** | **.06** | **.00** | **.06** | **.07** | **< .001** |
| Female gender | .05 | .03 | -.01 | -.11 | .088 |
| **Low education** | **.39** | **.04** | **.31** | **.47** | **< .001** |
| **Medium education** | **.12** | **.04** | **.05** | **.19** | **.001** |
| High education | b. | --- | --- | --- | --- |
| Cohabitating (Yes) | -.03 | .04 | -.10 | .04 | .420 |
| Widowhood | .02 | .04 | -.06 | .11 | .591 |
| Urban living environment | -.02 | .03 | -.08 | .04 | .499 |
| **Loneliness** | **.08** | **.01** | **.06** | **.10** | **< .001** |
| **Lack of motivation** | **.14** | **.02** | **.10** | **.18** | **< .001** |
| Social network size | -.00 | .01 | -.02 | .01 | .663 |
| **Sum social out-of-home activities** | **-.15** | **.02** | **-.18** | **-.11** | **<.001** |
| Increase of **social out-of-home-activities** SHARE Wave 6 to Wave 7 | **-.22** | 0.4 | **-.29** | **-.14** | **< .001** |
| Note: **n** = 17,439; SHARE = Survey of Health, Aging, and Retirement in Europe; SE=standard error  LCI = lower confidence interval; UCI = upper confidence interval; b.=reference category; model controlled for frailty status at baseline; country dummy variables were entered as random effect | | | | | |
