# Supplementary material for: Prevention of frailty in relation with social out-of-home activities in older adults: results from the Survey of Health, Ageing, and Retirement in Europe
Source: Eur J Ageing. 2024 Nov 16;21(1):35. doi: 10.1007/s10433-024-00829-7 (PMC11569102; doi:10.1007/s10433-024-00829-7)
Supplement: Supplementary file 3 — (DOCX 38 kb) [file 10433_2024_829_MOESM3_ESM.docx]

Appendix 3

Results of the sensitivity analyses of the fully adjusted cross sectional mixed model on frailty status in SHARE wave 6

| Predictor | β | SE | 95 % LCI | 95 % UCI | *P* value |
| --- | --- | --- | --- | --- | --- |
| SHARE Wave 6 (2015) |  |  |  |  |  |
| **Age** | **.05** | **.00** | **.04** | **.05** | **< .001** |
| Female gender | **.28** | **.03** | **.23** | **.34** | **< .001** |
| **Low education** | **.71** | **.04** | **.64** | **.79** | **< .001** |
| **Medium education** | **.30** | **.03** | **.23** | **.36** | **< .001** |
| High education | b. | -- | -- | -- | -- |
| Cohabitating (yes) | .00 | .03 | -.07 | .07 | .954 |
| Widowhood | .06 | .04 | -.02 | .14 | .153 |
| Urban living environment | -.03 | .03 | -.09 | .03 | .317 |
| **Loneliness** | **.36** | **.01** | **.33** | **.38** | **< .001** |
| **Lack of motivation** | **.73** | **.02** | **.69** | **.77** | **< .001** |
| **Social network size** | **.05** | **.01** | **.03** | **.07** | **< .001** |
| **Sum social out-of-home activities** | **-.18** | **.02** | **-.21** | **-.15** | **< .001** |
| Note: **n = 17,439**; SHARE = Survey of Health, Aging, and Retirement in Europe; SE=standard error  LCI = lower confidence interval; UCI = upper confidence interval; b.=reference category; model controlled for participant´s frailty status at wave 6, and participant´s county (dummy variables) as random effect | | | | | |

Results of the sensitivity analyses of the fully adjusted cross sectional mixed model on
frailty status in SHARE wave 7

| Predictor | β | SE | 95 % LCI | 95 % UCI | *P* value |
| --- | --- | --- | --- | --- | --- |
| SHARE Wave 7 (2017) |  |  |  |  |  |
| **Age** | **.07** | **.00** | **.06** | **.07** | **< .001** |
| Female gender | **.34** | **.05** | **.23** | **.44** | **< .001** |
| **Low education** | **.50** | **.07** | **.35** | **.64** | **< .001** |
| **Medium education** | **.11** | **.07** | **-.03** | **.25** | **< .001** |
| High education | b | -- | -- | -- | -- |
| Cohabitating (yes) | -.10 | .07 | -.24 | .03 | .121 |
| Widowhood | -.04 | .08 | -.19 | .11 | .566 |
| Urban living environment | .06 | .06 | -.18 | .05 | .195 |
| **Loneliness** | **.36** | **.02** | **.32** | **.41** | **< .001** |
| **Lack of motivation** | **.80** | **.04** | **.73** | **.87** | **< .001** |
| Social network size | **--** | **--** | **--** | **--** | -- |
| **Sum social out-of-home activities** | **-.27** | **.04** | **-.34** | **-.19** | **< .001** |
| Increase of social out-of-home-activities SHARE Wave 6 to Wave 7 | .06 | .08 | -.10 | .22 | .457 |
| Note: **n = 4767**; due to missing values about participants frailty status n=12,407,urban living environment n=455, loneliness n=12,434 and lack of motivation n=12,5774 in SHARE wave 7 the sample size was reduced; SHARE = Survey of Health, Aging, and Retirement in Europe; Social network Size was not included in SHARE wave 7; SE=standard error LCI = lower confidence interval; UCI = upper confidence interval; b.=reference category;; model controlled for participant´s frailty status at wave 6, and participant´s county (dummy variables) as random effect | | | | | |

Results of the sensitivity analyses of the fully adjusted cross-sectional mixed model on frailty status in SHARE wave 8

| Predictor | β | SE | 95 % LCI | 95 % UCI | *P* value |
| --- | --- | --- | --- | --- | --- |
| SHARE Wave 8 (2020) |  |  |  |  |  |
| **Age** | **.07** | **.00** | **.06** | **.07** | **< .001** |
| Female gender | **.26** | **.03** | **.19** | **.32** | **< .001** |
| **Low education** | **.71** | **.04** | **.63** | **.80** | **< .001** |
| **Medium education** | **.26** | **.04** | **.18** | **.33** | **< .001** |
| High education | b. | -- | -- | -- | -- |
| Cohabitating (yes) | -.07 | .04 | -.14 | .01 | .147 |
| **Widowhood** | **.10** | **.05** | **.01** | **.19** | **.027** |
| Urban living environment | -0.6 | .03 | -.13 | .01 | .101 |
| **Loneliness** | **.39** | **.01** | **.38** | **.41** | **< .001** |
| **Lack of motivation** | **.78** | **.21** | **.74** | **.82** | **< .001** |
| Social network size | **.05** | **.01** | **.03** | **.06** | **< .001** |
| **Sum social out-of-home activities** | **-.27** | **.02** | **-.31** | **-.24** | **< .001** |
| Increase of social out-of-home-activities SHARE Wave 6 to Wave 7 | -.08 | .04 | -.16 | .00 | .054 |

Note: **n = 15,880**; due to missing values about participants urban living environment n=698, loneliness n=225, lack of motivation n=739 and sum of social out-of-home activities n= 214 in SHARE wave 8 the sample size was reduced SHARE = Survey of Health, Aging, and Retirement in Europe; SE=standard error; LCI = lower confidence interval; UCI = upper confidence interval; b.=reference category; Social network Size was not included in SHARE wave 7; model controlled for participant´s frailty status at wave 6, and participant´s county (dummy variables) as random effect

Results of the sensitivity analyses of the fully adjusted mixed model on the change of frailty measured with the Edmonton Frail Scale between SHARE wave 6 and 7

| Predictor | β | SE | 95 % LCI | 95 % UCI | *P* value |
| --- | --- | --- | --- | --- | --- |
| SHARE Wave 6 (2015) |  |  |  |  |  |
| **Age** | **.04** | **.00** | **.04** | **.05** | **< .001** |
| **Female gender** | **.12** | **.05** | **.03** | **.21** | **.010** |
| **Low education** | **.15** | **.06** | **.03** | **.27** | **.020** |
| Medium education | -.02 | .06 | -.14 | .09 | .683 |
| High education | b. | -- | -- | -- | -- |
| Cohabitating (yes) | -.05 | .06 | -.16 | .06 | .397 |
| Widowhood | -.12 | .07 | -.27 | .01 | .069 |
| Urban living environment | .02 | .05 | -.08 | .11 | .482 |
| **Loneliness** | **.10** | **.02** | **.07** | **.14** | **< .001** |
| **Lack of motivation** | **.19** | **.03** | **.13** | **.25** | **< .001** |
| Social network size | .02 | .01 | -.01 | .05 | .121 |
| **Sum social out-of-home activities** | **-.16** | **.03** | **-.21** | **-.10** | **< .001** |
| **Increase of social out-of-home-activities SHARE Wave 6 to Wave 7** | **-.27** | **.06** | **-.39** | **-.15** | **< .001** |
| Note: **n = 5032** ; due to missing values about participants frailty status in SHARE  wave 7 n=12,407 the sample size was reduced; SHARE = Survey of Health, Aging, and Retirement in Europe; SE=standard error; LCI = lower confidence interval; UCI = upper confidence interval; b.=reference category; Social network size was not included in SHARE wave 7; model controlled for participant´s frailty status at wave 6, and participant´s county (dummy variables) as random effect | | | | | |
